# Supplementary material for: Complex changes in serum protein levels in COVID-19 convalescents
Source: Sci Rep. 2024 Feb 23;14:4479. doi: 10.1038/s41598-024-54534-7 (PMC10891133; doi:10.1038/s41598-024-54534-7)
Supplement: Supplementary file 1 — Supplementary Information 1. [file 41598_2024_54534_MOESM1_ESM.pdf]

Supplementary materials

Supplementary Information 2.

The file contains demographic data, information on protein level measurements and analysis, as well as order of the mass spectrometry experiments.

Supplementary Information 3.

The file contains information on peptide level measurements, including post-translational modifications, and analysis of hexose modified peptides.

Table S1: Demographics of COVID-19 convalescents.

Demographic data per healthy and convalescent subject are provided in **Supplementary Information 2.**
IQR - interquartile range

**Characteristics**		**COVID-19 convalescents (n=29)**		**p-value**
	Total in COVID-19 convalescents cohort	Symptomatic	Asymptomatic	
**Sex, Age years, median (IQR)**	44 (20)			
Male, n=16 (55%)	45 (21)	7 (24%)	8 (28%)	0.69
Female, n=13 (45%)	43 (20)	8 (28%)	5 (17%)	
**Race (% of total)**				
White	9 (31%)	5 (17%)	4 (14%)	
African American	17 (59%)	9 (31%)	8 (28%)	
Hispanic	2 (7%)		1 (3%)	
Asian	1 (3%)	1 (3%)		
**Antibody Response Titer, median (IQR)**		4,050 (8,100)	675 (1,349)	<0.01
No antibody response	8 (28%)			
Low (1:450)	1 (3%)			
Moderate (1:1,350)	6 (21%)			
High (1:4,050)	6 (21%)			
Very high (1:12,150 or higher)	7 (24%)			
**Days since diagnosis, median (IQR)**		59 (15)	32 (8)	<0.001
**Symptoms/no symptoms, n (%)**		15 (52%)	13 (45%)	
Fever		9 (31%)		
Shortness breath		7 (24%)		
Loss of taste		6 (21%)		
Cough		6 (21%)		
Loss of smell		5 (17%)		
Back pain		4 (14%)		
Fatigue/weakness		4 (14%)		
Chills		3 (10%)		
Sore throat		2 (7%)		
Stomach/abdominal pain		2 (7%)		
Hypertension		1 (3%)		
Labored breathing		1 (3%)		
Loss of appetite		1 (3%)		
Dizziness		1 (3%)		
Congestion		1 (3%)		
Sweating		1 (3%)		
Gastrointestinal		1 (3%)		
Bilateral pneumonia		1 (3%)		
Itchy eyes		1 (3%)		
Running nose		1 (3%)		
Chest pain		1 (3%)		
Aches		1 (3%)		

Figure S1. Quality control: fragment intensity variation for quality control samples

We analyzed pooled quality control (QC) samples along with cohort examples (every 6-10 samples). Data was acquired in four sets (batches) listed in **Supplementary Information 2.** To assess variability of the data, we examined the 334 proteins we discuss in the main text for their variability across the QC samples (labeled ‘Upool’ in the figure) . All data was normalized within and across batches (sets) as described in the **Methods**.

A. Antibody titer levels amongst COVID-19 convalescents (Titer as 1:X) and Days since Diagnosis show substantial correlation.

B. The four histograms show the distribution of the Coefficients of Variance (CoV) for the protein levels grouped into the four technical batches. Almost all proteins (330 of 334) have <50% CoV in at least one set which is in the expected range for untargeted (shotgun) proteomics. We did not filter for CoV but marked the few proteins of CoV>50% in **Figure 3B** and in the main text.

C. The plot shows all samples, including the QC samples (‘Upool’), after normalization in their distribution across the first two principal components. This figure is analogous to **Figure 3A**. The Healthy controls and COVID-19 convalescents are clearly separated and QC samples cluster in the center. A minor exception are QC samples from technical batch 3 which group locate off center; however COVID-19 convalescents and Healthy control samples from batch 3 cluster correctly with their respective groups, indicating successful data acquisition and normalization.

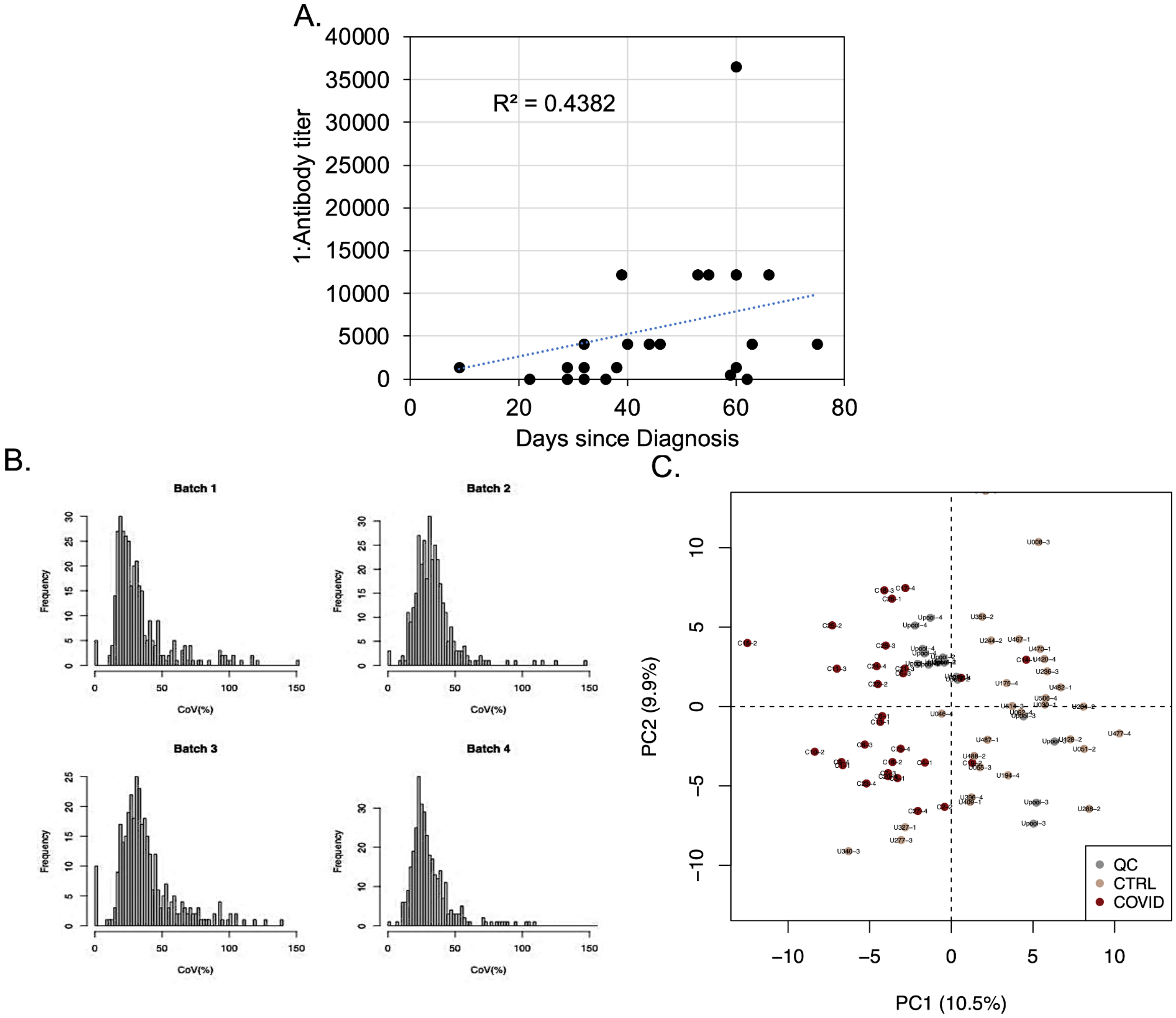

Figure S2. Quality control: expected expression levels of female-specific proteins

Heatmap showing levels of Pregnancy Zone Protein (PZP) along with proteins with similar patterns as well as Lactotransferrin (LTF).

A. The panel shows the color-coded protein levels for female individuals from both cohorts.

B. The panel shows the significance values for different comparisons and different models. Significance values (adjusted p-values) were transformed as follows: if the observed $\log_{10}$-transformed level fold change was positive, we calculated 1-[adjusted p-value]; if negative, we calculated -(1-[adjusted p-value]). Dark colors indicate adjusted p-value <0.05; light colors adjusted p-value < 0.20. Columns represent select comparisons: the Overall difference between COVID-19 convalescents and healthy controls; the role of sex and race in a multivariate model in which other factors such as age were also considered; and the role of Days since diagnosis and the presence of Symptoms in a univariate model which considered only one factor at a time. Each statistical model was developed separately for the factor in question and the respective sample set: healthy controls (beige), COVID-19 convalescents (brown); $\log_{10}$-transformed ratio of paired protein levels for COVID-19 cases and healthy control (beige-brown striped). The complete results of the statistical testing are provided in **Supplementary Information 2**.

Importantly, PZP is the only protein of the 334 total proteins with a sex difference: it has slightly higher levels in healthy women than inhealthy men (adjusted p-value < 0.20). As PZP levels are thought to associate with pregnancy, the results suggest that some of the female individuals were pregnant, causing the difference in levels between the two sexes. Further, while not significant, LTF shows a general decline in levels with age of healthy female individuals, concurrent with the role of LTF in breastmilk.

POC - person of color

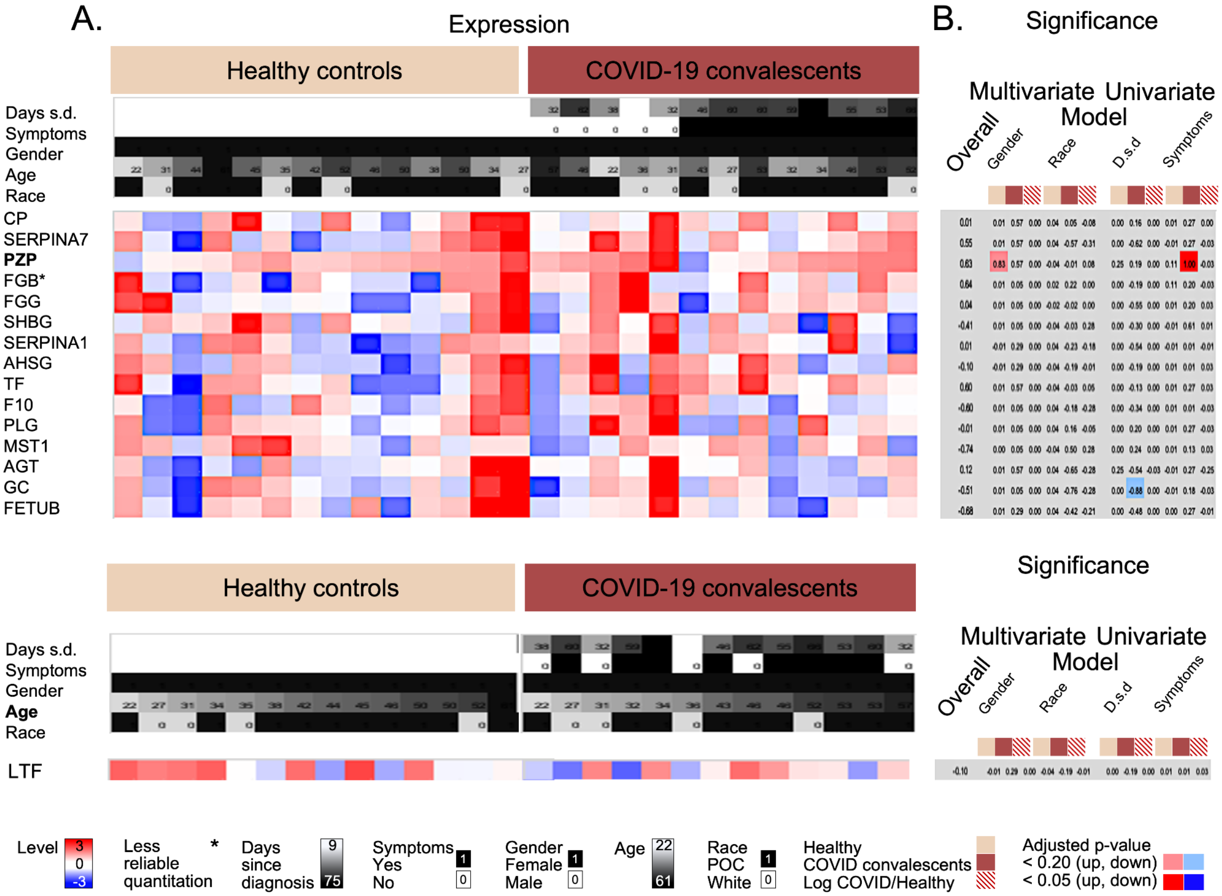

Figure S3. Quality control: expected expression patterns of obesity related proteins

The figure shows the meta data and protein levels for IGFALS and IGF1 for 5 healthy control individuals. Both proteins have been connected to obesity. The 5 individuals were selected based on similar age (50 to 53 years) but different body mass index (BMI): normal weight (BMI<25) versus obese (BMI>30). Due to the small size of the cohort, there were no additional sets of age-matched individuals from different BMI categories. We found a significant difference in IGFALS and IGF1 levels between normal weight and obese individuals (P-value , 0.05, two-sample t-test). The demographic information and protein level data are provided in **Supplementary Information 2.**

POC - person of color

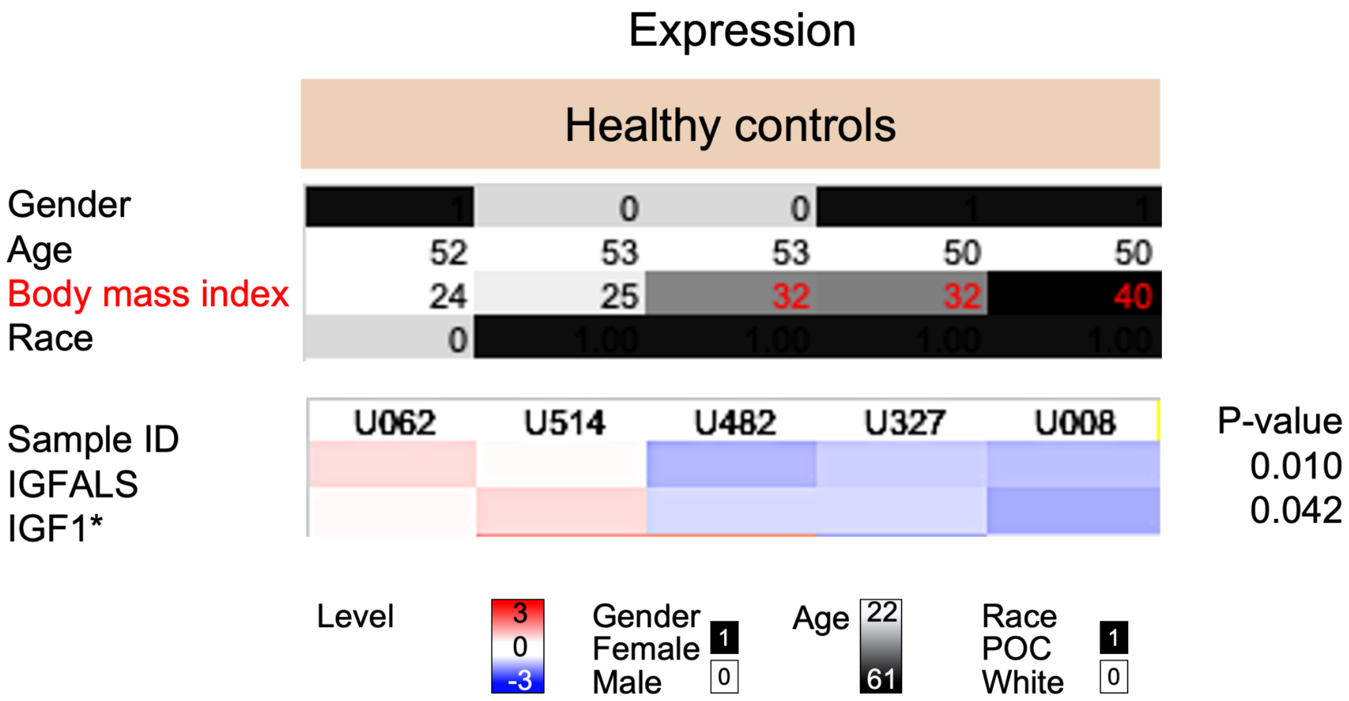

Figure S4. Proteins with statistically different levels between COVID-19 convalescents and healthy controls

Shown are the normalized protein levels for the statistically significantly expressed proteins (adjusted p-value < 0.05). All data is available in **Supplementary Information 2**. Samples are organized according to sex and age. Proteins with high Coefficient of Variance are marked with * (>50%). POC - person of color; S.d. - since diagnosis.

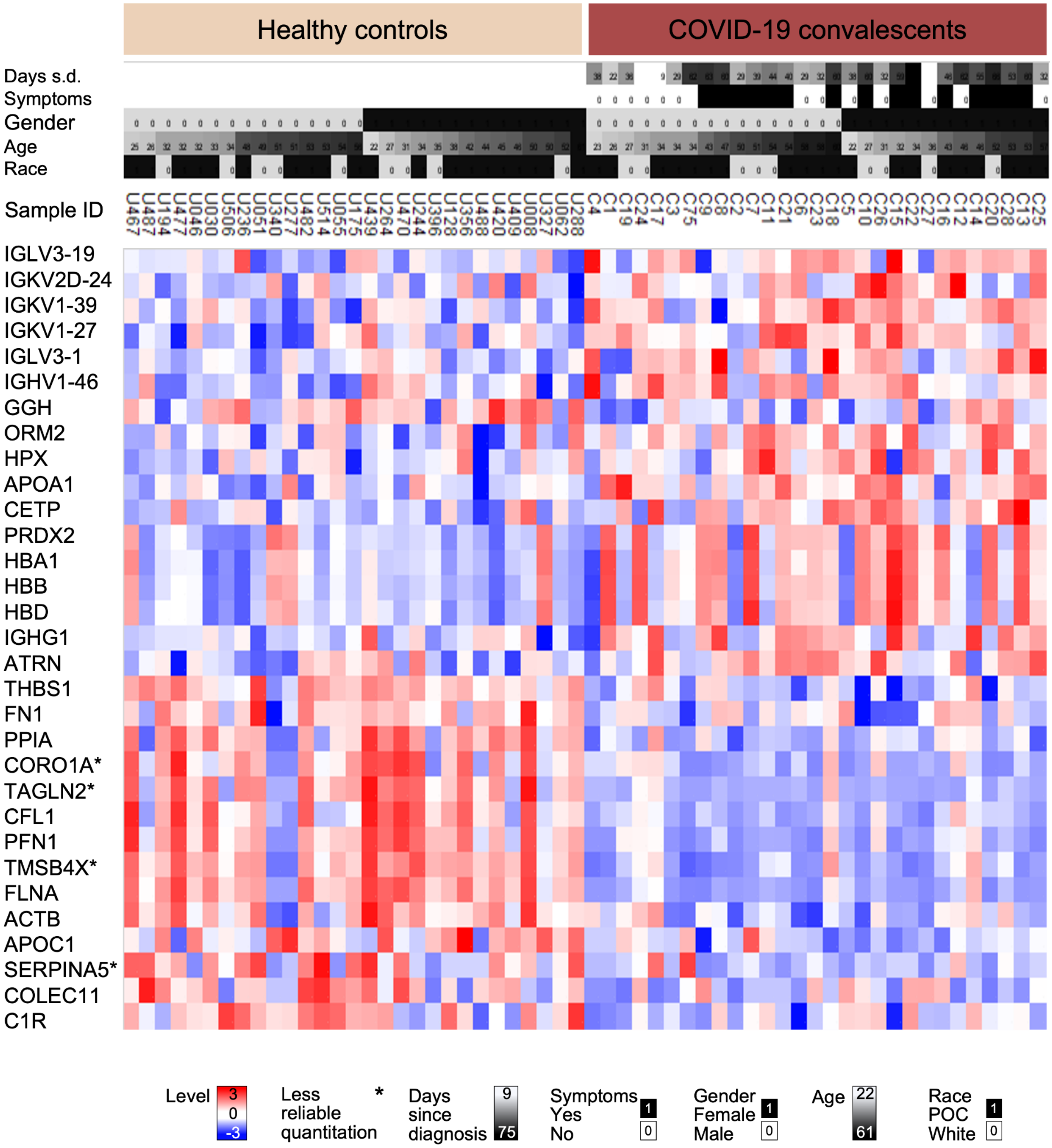

Figure S5. Proteins whose levels were similar between COVID-19 convalescents and healthy controls

Heatmap showing proteins from three pathways that are largely similar in healthy controls and COVID-19 convalescents.

A. The panel shows the color-coded protein levels with samples sorted according to sex and age.

B. The panel shows the significance values (associations) for different comparisons and different models. Significance values (adjusted p-values) were transformed as follows: if the observed $\log_{10}$-transformed fold change was positive, we calculated 1-p; if negative, we calculated -(1-p). Dark colors indicate adjusted p-value <0.05; light colors adjusted p-value < 0.20; grey: no significance. Columns represent select comparisons: the 'Overall' difference between COVID-19 convalescents and healthy controls; and the impact of Days since diagnosis, the presence of Symptoms, Sex, Age, and Race in a multivariate model using the healthy control (beige) or COVID-19 convalescents (brown). The complete results of the statistical testing are provided in **Supplementary Information 2**.

Clusters: 1 Coagulation cascade; 2 Complement system; 3 Inflammation.

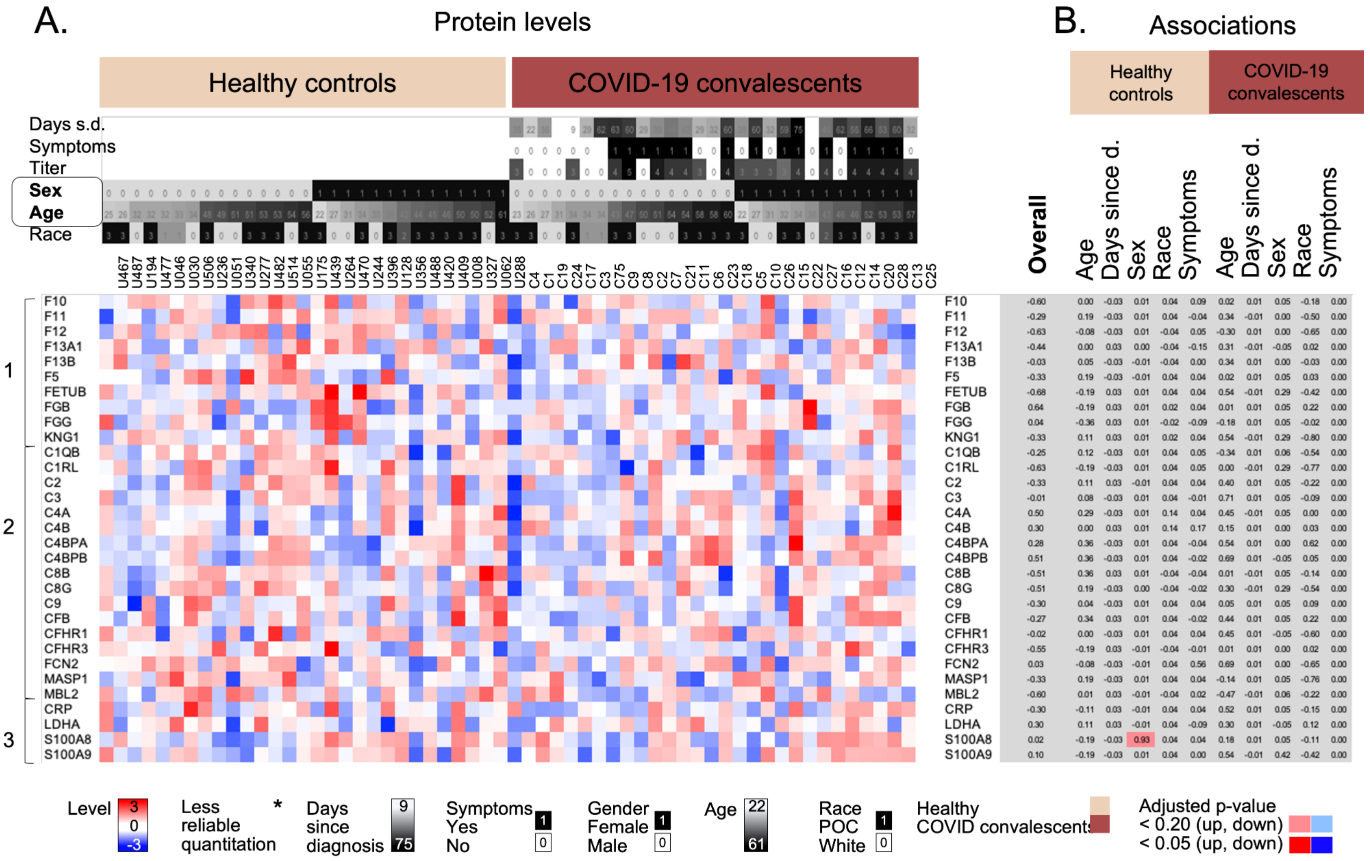

Figure S6. Post-translational modifications

The heatmap shows examples of normalized levels of hexose modified peptides. Three peptides (two for albumin (ALB) and one for Immunoglobulin heavy constant alpha 1 (IGHA1) were significantly more modified amongst COVID-19 convalescents compared to healthy controls (adjusted p-value < 0.05) . The heatmap shows additional modified peptides with an adjusted p-value < 0.20. For visualization, data was row median centered and row Z-score normalized. While highly heterogeneous, healthy controls show more often low levels of modification than COVID-19 convalescents, in particular amongst women. The complete results are provided in Supplemental Information 3.

The peptide sequence is shown in the key below the heatmap.

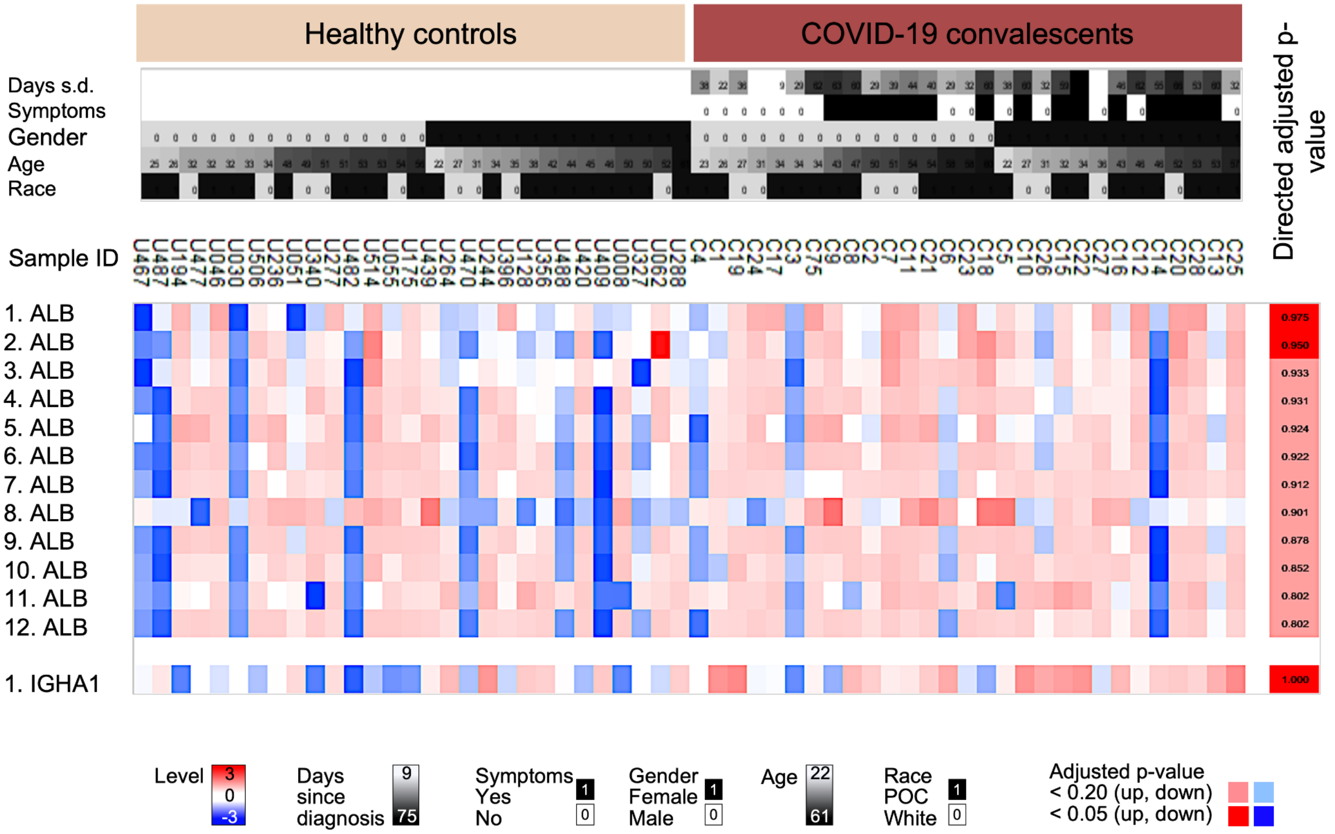

Key:
1. ALB - AFK[Hex]AWAVAR
2. ALB - K[Hex]LVAASQAALGL
3. ALB - K[Hex]YLYEIAR
4. ALB - CASLQK[Hex]FGER
5. ALB - NYAEAK[Hex]DVFLGMFLYEYAR
6. ALB - FPK[Hex]AEFAEVSK
7. ALB - LDELR[Hex]DEGKASSAK
8. ALB - VTK[Hex]CCTESLVNR
9. ALB - EQLK[Hex]AVMDDFAAFVEK
10. ALB - LDELRDEGK[Hex]ASSAK
11. ALB - SHCAEVENDEMPADLPS[Hex]LAADFVESKDVCK
12. ALB - DVCK[Hex]NYAEAK

1. IGHA1 - GDTFS[Hex]CM[Oxidation (M)]VGHEALPLAFTQK
